# Retinal and Choroidal Vasculature in Patients with Marfan Syndrome

**DOI:** 10.1167/tvst.9.9.5

**Published:** 2020-08-04

**Authors:** Matteo Di Marino, Massimo Cesareo, Gianluca Aloe, Carlo Nucci, Clarissa Giannini, Alessio Martucci, Francesco Aiello, Calogera Pisano, Giovanni Ruvolo, Raffaele Mancino

**Affiliations:** 1 Ophthalmology Unit, Department of Experimental Medicine, University of Rome Tor Vergata, Rome, Italy; 2 Department of Cardiac Surgery, Tor Vergata University Hospital of Rome, Rome, Italy

**Keywords:** OCTA, OCT, Marfan syndrome, retina, choroid

## Abstract

**Purpose:**

To assess the retinal and choroidal vasculature in patients with genetically confirmed Marfan syndrome (MfS).

**Methods:**

This prospective, case-control, observational study included 48 eyes of 24 patients with a genetic diagnosis of MfS and compared them with 52 eyes of 26 healthy controls. Best-corrected visual acuity, choroidal and retinal thickness measured by spectral domain-optical coherence tomography, retinal and choroidal vasculature characterized by optical coherence tomography angiography, were collected. A genetic counseling was carried out. A transthoracic echocardiogram was performed to evaluate the dimension of the aortic root, the ascending aorta and the left ventricle function and dimensions.

**Results:**

A significant decrease in the superficial and deep retinal capillary plexi vessel density (VD) was evident, such as a decrease in the choriocapillaris plexus VD. In patients with MfS, a negative correlation between left ventricular diameter and the VD of the superficial and deep plexi was observed. Patients with MfS with greater posterior wall and interventricular septum dimensions had lower VD in both plexi (*P* < 0.05). Moreover, there was a negative correlation between the dimension of the ascending aorta and foveal choriocapillary VD. In patients with MfS, increasing diameter of the ascending aorta was associated with a lower foveal choriocapillary VD (*P* < 0.05).

**Conclusions:**

The severity of MfS correlates with the impairment of the retinal and choroidal vasculature.

**Translational Relevance:**

Optical coherence tomography angiography may be a reproducible and noninvasive tool to study retinal blood flow in patients with MfS, with potential diagnostic and prognostic value.

## Introduction

The Marfan syndrome (MfS) is a genetic autosomal dominant disorder of connective tissue that involves multiple systems, including the eye.^1^ Many affected individuals have a mutation in the gene that codes for fibrillin-1 (FBN1), located on chromosome 15q21.1. Fibrillin is a widely expressed glycoprotein found in elastic fibers of the aorta and nonelastic tissues, such as ciliary zonules of the eye, the periosteum of bones, and tendons.

The diagnosis is usually clinical, requiring fulfilment of certain criteria according to Ghent nosology, including a wide spectrum of systemic features (mainly cardiovascular, skeletal, and ocular), family history, and FBN1 mutation.^2^ In the 2010 revision of the Ghent nosology, ectopia lentis resulting from instability and rupture of the ciliary zonule, represents one of the major criteria and the most common ocular sign (>60%). Other ocular features included an axial myopia of more than 3 diopters, microspherophakia, cataract, glaucoma, iris transillumination defects, and a flattened cornea.^3^^−^^8^

Our study evaluated macular and optic nerve vessel density (VD) of patients with genetically confirmed MfS using optical coherence tomography angiography (OCTA). We also investigated the mean choroidal thickness and central retinal thickness of these patients’ eyes with spectral domain OCT. A transthoracic echocardiogram was performed to evaluate the dimension of the aortic root, the ascending aorta and the left ventricle function and dimensions. All correlations between echocardiographic and eye variables were investigated.

Our hypothesis is that the fundamental aortic and cardiovascular abnormalities characteristics of the MfS may result in reduced retinal and choroidal VD. It is reasonable that the death from apoptosis of the vascular smooth muscle cells demonstrated in the MfS could also occur at the level of the ocular vascular networks.^9^ This would result in an alteration of the VD detectable with OCTA.

This method does not directly measure the extent of retinal flow, but can be considered a surrogate biomarker; in fact, it makes only vessels inhabited by a certain amount of blood flow visible.

## Methods

In this prospective, case-control, observational study, 24 patients with a genetic diagnosis of MfS were compared with 26 healthy controls (HC) similar for age and sex. All participants gave their informed consent according to the Declaration of Helsinki. The study was approved by the internal review board at the University Hospital of “Tor Vergata,” Rome (Protocol Number:179/18).

Twenty-two patients with MfS (95.7%) presented a FBN1: NM-000138 gene mutation, one patient had a FBN1: NM-000139 gene mutation, and a single individual did not undergo genetic analysis. We identified four different types of mutations occurring with varying incidence: FS (15.4%), MISSENSE (7.7%), MISSENSE CYS (15.4%), SPLICING (53.9%), STOP (7.7%), and MISSING (45%) (Table 1).

**Table 1.** Genetic Variables

Genetic Variables	No. (%)
Gene	
*FBN1: NM_000138*	22 (95.7)
*FBN1: NM_000139*	1 (4.3)
Missing	1
Type of mutation	
*FS*	2 (15.4)
*MISSENSE*	1 (7.7)
*MISSENSE CYS*	2 (15.4)
*SPLICING*	7 (53.9)
*STOP*	1 (7.7)
*MISSING*	11 (45)

All patients with MfS underwent transthoracic echocardiography on admission to evaluate the dimensions of the aortic root and the ascending aorta, and the function and dimension of the left ventricle. Echocardiography was performed by the same expert cardiologist in all patients. Evaluation of ascending aorta diameter was performed as follows: dimensions of the aortic annulus, sinuses of Valsalva, and proximal ascending aorta (above 2.5 cm of the sinotubular junction) were estimated in the parasternal long axis view, and the aortic arch was evaluated using a suprasternal view. Echocardiography-derived dimensions were reported as internal diameter size. Color Doppler imaging was used to assess the presence and severity of aortic regurgitation and stenosis.^10^

Aortic root diameter Z-scores were calculated using Gautier's formula. Z-scores are a means of expressing the deviation of a given measurement from the size-specific population mean. Aortic root dilatation was defined as any aortic root measurement Z-score of 2 or greater at the sinus of Valsalva, at the sinotubular junction, or at the level of the ascending aorta. The rate of aortic dilatation was defined as the ratio between the difference of measured aortic diameters and the interval, in years, between the two examinations. Because of the natural dilation of the aortic root in children, a threshold of maximum dilation rate above the 90th percentile (5 mm/y) was set as being clinically relevant. The difference between the first and last sinus of Valsalva Z-scores available during follow-up was calculated and was divided by the delay between the two measurements to obtain the sinus of Valsalva Z-score evolution rate during the follow-up period.^11^

All patients underwent full ophthalmologic examinations including best-corrected visual acuity, spectral domain OCT (Spectralis; Heidelberg Engineering, Heidelberg, Germany), and OCTA (Avanti AngioVue Imaging System; Optovue, Inc., software version 2017.1.0.26, Fremont, CA).

To evaluate the mean choroidal thickness, two enhanced depth imaging OCT subfoveal scans were performed in all patients and in the HC group by two independent observers. The choroidal thickness was measured subfoveally, 1000 µm nasally and temporally to the fovea. The average value was then obtained and used in the statistical analysis. Choroidal thickness was measured from the outer portion of the hyperreflective line corresponding with the retinal pigment epithelium to the hyporeflective line corresponding with the sclerochoroidal interface. The central retinal thickness was similarly obtained.^12^^,^^13^

Both eyes of each participant were examined with a 6 × 6-mm scanning protocol of the macula and with a 4.5 × 4.5-mm scanning protocol of the optic nerve using Avanti Angiovue OCTA. The VD was then calculated using the instrument's built-in software. We analyzed the VD of the superficial, deep and choriocapillary plexus of the macula, and the radial peripapillary capillary network of the optic nerve. In addition, retinal nerve fiber layer measures were obtained by the same scanning protocol. Poor image quality according to specific criteria including low quality index (<7), presence of blink artifacts, motion or doubling artifacts caused by poor fixation, and media opacities obscuring the view of the vasculature were considered exclusion criteria. All examinations were analyzed by the same expert ophthalmologist.

Exclusion criteria for patients with MfS were the presence of any systemic or ocular disease with the exception of MfS, previous intraocular surgery with the exception of phacoemulsification, current use of any drug therapy known to be toxic to the retina and/or optic nerve, and spherical refractive errors higher than 6 diopters. Both MfS and HC groups presented a best-corrected visual acuity of 0.0 logarithm of the minimum angle of resolution or greater.

All nonquantitative variables are reported as frequencies and percentages, and quantitative variables have been synthesized as mean and standard or median deviation with minimum and maximum. To compare the patient group with MfS and the HC group, the *t*-test, the Welch test, or the Wilcoxon test for quantitative variables were calculated after checking normality and homoscedasticity assumptions. In addition, the Pearson correlation coefficient was calculated to evaluate possible correlations between echocardiographic and eye variables.

The analyses were processed using the STATA 14.1 software (StataCorp, College Station, TX) and all tests were considered significant at a *P* value of less than 0.05.

## Results

There were no statistical differences between the two groups (*P* > 0.05) (Table 2). Echocardiographic features are summarized in Table 3.

**Table 2.** Demographic Characteristics

	MfS (*n* = 24)	HC (*n* = 26)	*P* Value
Age	29 [18–64]	29 [20–70]	0.876
Height	175 [133–195]	173 [159–194]	0.439
Weight	67.6 ± 24.9	69.2 ± 12.8	0.786
Body mass index	21.5 [11.0–41.3]	22.3 [18.4–37.2]	0.185

**Table 3.** Echocardiographic Features

Echocardiographic Features	MfS	HC	*P* Value
BSA Dubois	1.8 ± 0.4	1.8 ± 0.2	0.659
Anulus	23.2 ± 4.8	19.8 ± 3.5	0.038
Aortic root	43 [23 to 61]	33 [24 to 36]	0.000
Arco	20 [17 to 31]	20 [18 to 31]	0.422
AO abdominal	15 [10 to 24]	13 [10 to 16]	0.129
AO ascending	30 ± 9.2	26.9 ± 3.2	0.253
AO descending	18 [12 to 26]	14 [13 to 26]	0.193
Sinotubular junction	30 [16 to 53]	27 [20 to 30]	0.048
Z score	3.3 [0.0 to 8.8]	0.3 [–2.0 to 1.4]	<0.001
Ratio	1.4 [1 to 2]	1 [0.8 to 1.2]	<0.001
Left ventricle end-diastolic volume	48 [25 to 63]	47 [25 to 56]	0.231
Left ventricle end-systolic volume	30 [17 to 78]	31.5 [15 to 41]	0.717
Posterior wall	9.1 ± 1.4	8.7 ± 2	0.422
Septum	9.1 ± 1.7	8.3 ± 1.8	0.089

Ao, aorta; BSA, body surface area.

Patients with MfS presented significantly different values for dilation of the aortic root, sinotubular junction and ascending aorta compared to HC (*P* < 0.005). In patients with MfS, there was a negative correlation between left ventricular diameter and the VD of the superficial and deep plexus. In particular, patients with increased thickness of the posterior wall and interventricular septum demonstrated lower VD both of the superficial and deep plexus (*P* < 0.05). Similarly, a negative correlation was found between the diameter of the ascending aorta and the foveal choriocapillary VD. Patients with MfS with a more dilated ascending aorta had lower foveal choriocapillary VD (*P* < 0.05). In contrast, no statistically significant correlation was observed between diameter of the bulbus aortae and ophthalmic parameters (*P* > 0.05). The central retinal thickness and the choroidal thickness were not significantly different between the two groups (*P* > 0.05). The VD of both superficial and deep plexi were significantly decreased in patients with MfS, with the deep plexus particularly affected (*P* < 0.05) (Figure). VD defects in the choriocapillaris plexus were made evident, and a significant enlargement of the foveal avascular zone was also found. No statistically significant difference emerged from the analysis of radial peripapillary capillary values (*P* > 0.05). All data are summarized in Table 4.

**Figure.**
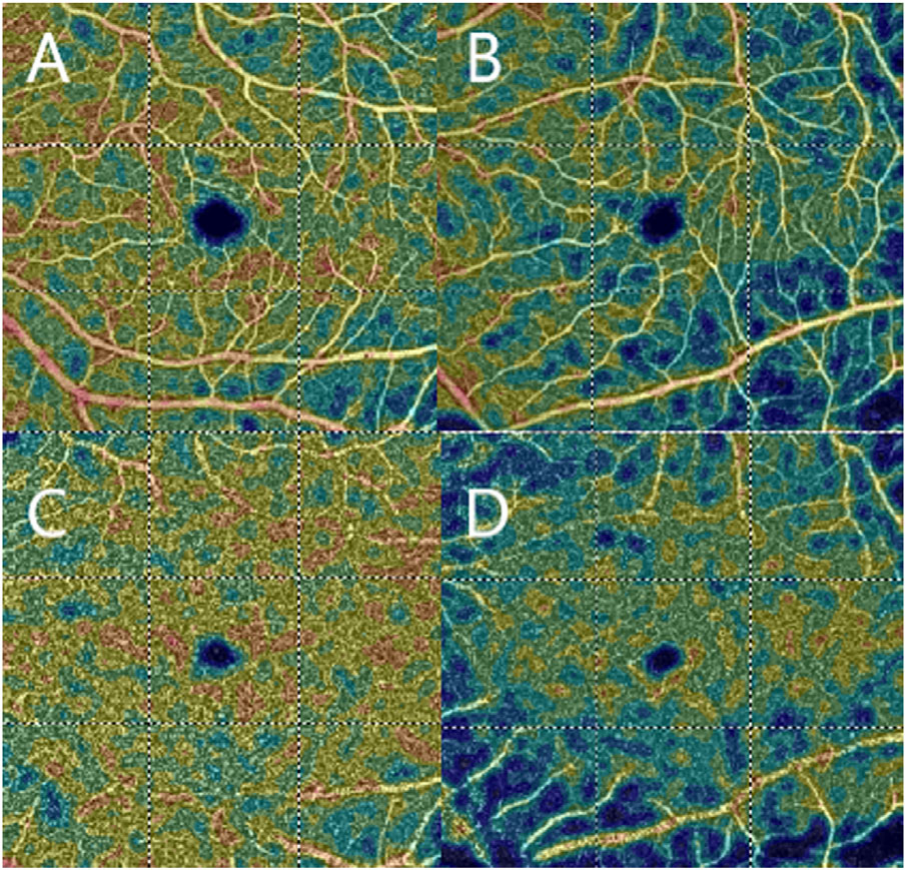
OCTA scans. (A) HC superficial plexus. (B) MfS superficial plexus. (C) HC deep plexus. (D) MfS deep plexus.

**Table 4.** Ophthalmic Parameters

Ophthalmic Parameters	MfS	HC	*P* Value
Mean choroidal thickness	306.8 [133–566]	304.7 [129.7–455]	0.434
Superficial plexus VD	49.8 [38.1–55.7]	52.6 [47.8–56.5]	<0.001
Superficial fovea VD	22.4 ± 7.1	26.2 ± 7.2	0.017
Deep plexus VD	52.7 [30.3–61.9]	59.3 [42–65.8]	<0.001
Deep fovea VD	39.6 ± 8.1	45.5 ± 6.4	<0.001
Choriocapillary VD	73.2 [65.6–78.2]	75.7 [71.5–78.2]	0.006
Choriocapillary fovea VD	73.8 [43.1–79.4]	75.6 [68.2–81.2]	0.034
Foveal avascular zone	0.2 [0.1–0.5]	0.2 [0–0.3]	0.018
Radial peripapillary capillary	48.4 ± 2.5	48.4 ± 3.1	0.999

## Discussion

The fibrillins are extracellular matrix molecules that polymerize to form microfibrils in the connective tissue. There are three types in humans, with both structural and regulatory roles.^14^^,^^15^ The elasticity and the structural support of these molecules is fundamental in maintaining the stability of the extracellular matrix. The most studied genetic disorder associated with mutations in FBN1 is MfS.^16^ These mutations are distributed throughout the length of the *FBN1* gene, and lead to the degeneration of the elastic microfibrillar architecture.^9^^,^^17^ Several clinical manifestations are associated with this syndrome; cardiac, skeletal and ocular systems are often involved.^18^^–^^20^

OCTA is a relatively new, noninvasive imaging technique that allows for the visualization of blood vessels both in the retina and in the optic nerve head. This new tool has been successfully used to measure VD in the study of retinal vascular involvement in several systemic pathologies, such as multiple sclerosis and systemic lupus erythematosus.^21^^−^^26^

In this study, compromise of the retinal and choroidal vasculature was identified in patients with MfS.

The vascular smooth muscle cells, the most important cell type in the aortic wall in terms of blood flow regulation,^27^ die from apoptosis in patients with MfS, resulting in an immature aortic vessel wall.^28^^,^^29^ According to our data, the severity of cardioaortic parameters in MfS correlates with impairment of retinal blood flow. In fact, patients with a larger ascending aorta diameter and left ventricle hypertrophy displayed lower macular VD. This finding means that aortopathy in patients with MfS parallels retinal vascular defects. Interestingly, the VD of each central retina plexus (deep, superficial, and choriocapillaris) seemed to be significantly decreased, whereas the analysis of the radial peripapillary capillary showed no differences between the MfS and the HC groups. This finding could be at least in part due to the differing impact that large vessels of the optic disc have on the VD as a whole in these scans. Alternatively, this outcome could be linked to increased interindividual anatomic variability in the optic nerve, which makes this parameter less suitable in performing an objective comparison between the two groups.

However, to exclude any possible bias caused by differences in eye conformation in myopic patients, we selected only patients with a refractive error of less than 6 diopters and analyzed the choroidal thickness and central retinal thickness of both groups, which was not statistically different.

Recently, Xu et al.^3^ (2017) investigated the retinal nerve fiber layer thickness and central retinal thickness in a large cohort of patients with MfS using spectral domain OCT, demonstrating that 18% of patients with MfS presented a decrease in retinal nerve fiber layer thickness. These data could be related to abnormalities of the inner retinal layers, as a result of decreased blood flow. However, we did not find a statistical difference between retinal nerve fiber layer thickness in patients compared to HCs (*P* > .05).

To our knowledge, this is the first time that the retinal VD has been evaluated in patients with MfS. In recent years, the life expectancy of patients with MfS has improved significantly. Early diagnosis is pivotal in guaranteeing optimal management of these patients. Macular VD could represent a helpful biomarker supporting the diagnosis of MfS. Indeed, despite the fact that severity of aortic disease is the most important diagnostic and prognostic factor determining outcome in this disease, accurate measurements are often difficult to obtain. Some echocardiographic parameters are affected by increased interobserver and intraexamination variability,^30^^–^^32^ and recent studies have shown a high repeatability and reproducibility of OCTA examination. An OCTA evaluation is easy to perform, objective, and complication free,^33^^–^^35^ and could be a promising biomarker supporting diagnosis or in providing prognostic information. Pivotal OCTA data obtained from this relatively small cohort of patients with MfS may represent a promising biomarker supporting the diagnostic and/or prognostic process when used in a multidisciplinary approach that integrates omic, clinical, and instrumental findings into mathematical models.^36^ Future deep learning–based solutions could be able to use OCTA parameters with excellent accuracy, assisting clinicians in the categorization of rare pathologies.^37^
